# Health-related quality of life decreases in young people with asthma during the transition from adolescence to young adulthood: a birth cohort study

**DOI:** 10.1186/s12890-022-02259-6

**Published:** 2023-01-24

**Authors:** Maria Ödling, Niklas Andersson, Christer Janson, Erik Melén, Anna Bergström, Inger Kull

**Affiliations:** 1 Department of Clinical Science and Education, Södersjukhuset, Karolinska Institutet, Stockholm, Sweden; 2 Institute of Environmental Medicine, Karolinska Institutet, Stockholm, Sweden; 3 Department of Medical Sciences: Respiratory, Allergy and Sleep Research, Uppsala University, Uppsala, Sweden; 4 Sachs’ Children and Youth Hospital, Stockholm, Sweden; 5 Centre for Occupational and Environmental Medicine, Region Stockholm, Stockholm, Sweden

**Keywords:** Asthma control, Birth cohort, General health, Phenotype, Physical activity, Well-being

## Abstract

**Background:**

During the transition from paediatric to adult healthcare there is a gap between asthma guidelines and actual management with decreased healthcare consultations and dispensations of asthma medications after the transition to adult healthcare among young people with asthma. How health-related quality of life (HRQoL) develops during the transition from adolescence to young adulthood is unclear. Our aim was therefore to investigate HRQoL among young people with asthma during the transition to adulthood. Further, to assess if level of asthma control and physical activity influence any potential association between asthma and HRQoL.

**Methods:**

The study population consisted of 2268 participants from the ongoing Swedish population-based prospective birth cohort BAMSE (Barn/Child, Allergy, Milieu, Stockholm, Epidemiology). HRQoL was measured using the instrument EQ-5D-3 L and three general questions. The EQ-5D-3 L consists of the EQ-5D descriptive system and the EQ visual analogue scale (EQ VAS). The EQ-5D-3 L instrument and questions on general health, symptoms and treatment of asthma, and lifestyle factors were based on data from follow-ups at 16 and 24 years. Cross-sectional analyses were made.

**Results:**

At the 24-year follow-up, the adjusted median values of EQ VAS were lower compared with at the 16-year follow-up; among both participants with asthma (80 vs. 85, *p* < 0.01) and those without asthma (80 vs. 87, *p* < 0.01). At the 24-year follow-up, participants with uncontrolled asthma had a lower adjusted median EQ VAS score than peers with controlled/partly controlled asthma (75 vs. 80, *p* = 0.03). Further, young adults with asthma who did not fulfil the WHO recommendations on physical activity had lower EQ VAS scores than peers who did (70 vs. 80, *p* < 0.01).

**Conclusion:**

HRQoL is lower in young adulthood than in adolescence. Young adults with asthma having uncontrolled disease or who are physically inactive appear to be particularly vulnerable.

## Background

Adolescents and young adults are generally regarded as healthy age groups [1]. However, the development of asthma is a dynamic process, and asthma debuts in adolescence or young adulthood in some patients [2, 3]. An adolescent with a chronic disease like asthma experiences two types of transitions: moving from adolescence into adulthood and going from paediatric to adult healthcare [4, 5]. The latter transition should, according to asthma guidelines, be a well-planned and well-executed educational and therapeutic process [6]. However, in one of our recent studies, we investigated asthma-related healthcare consumption and pharmacological dispensation during the transition from paediatric to adult healthcare and found a clear gap between asthma guidelines and actual management [7]. Healthcare consultations were fewer than recommended in national guidelines and decreased even more after the transition to adult healthcare. Dispensations of asthma medications also decreased, even for participants with severe asthma, allergic asthma, and asthma in combination with airflow obstruction. In our research group we have also seen that in young adulthood the association between asthma and lower lung function was attenuated after adjustment for known risk factors in females in contrast to males [8]. Successful support during this period could provide young adults with lifelong skills for managing their asthma and thereby reduce the disease impact [1]. During childhood, including adolescence, living with asthma is associated with impaired HRQoL, especially if the asthma is uncontrolled [9, 10]. HRQoL, which encompasses physical function, social function, and mental health, is important for gathering a full view of the impact of the disease on patient wellbeing and provides a critical complement to clinical measures of disease state [11]. Previous studies have also showed that adolescent males with asthma report better HRQoL compared with female peers [12, 13]. Further factors in adolescence related to impaired HRQoL are chronic rhinosinusitis, food allergy, and being a current smoker [14–16]. However, how HRQoL develops during the transition to adulthood is unclear, as is the question of if the long-term goals of asthma treatment in achieving symptom control and maintaining normal activity levels have any influence. Reports of respiratory symptoms following physical exertion are common [2]. On the other hand, the World Health Organization (WHO) guidelines on physical activity and sedentary behaviour reaffirm that physical activity is good for health outcomes [17]. We therefore aimed to specifically investigate HRQoL during the transition to adulthood, among young people with asthma. Further, we aimed to assess if level of asthma control and physical activity influenced any potential association between asthma and HRQoL.

## Methods

### Study design and study population

During 1994–1996, parents to all new-borns living in predefined areas of Stockholm, the capital of Sweden, including inner city, urban, and suburban districts, were asked to participate in the population-based prospective birth cohort BAMSE (Barn/Child, Allergy, Milieu, Stockholm, Epidemiology) [18, 19]. The ongoing birth cohort includes 4,089 participants who have been followed since birth with repeated follow-ups. When the children were 2 months of age, parents answered a baseline questionnaire, providing information on covariates (sex, mother’s age at birth, parent born outside Sweden, family history of allergic disease, parental education, and parental smoking). When the participants were aged approximately 1, 2, 4, 8, 12 and 16 years, parents completed follow-up questionnaires to collect information about symptoms related to asthma and other allergic diseases, lifestyle factors and treatment of asthma. At 12, 16 and 24 years, participants were asked to complete questionnaires themselves. In addition to these questionnaires, the participants were invited to undergo clinical examinations when aged approximately 4, 8, 16 and 24 years.

This study population consisted of 2,268 participants (55.5% of the original cohort) who responded to the questionnaire and participated in the 24-year clinical examination (mean age when answering the questionnaire, 22.4 years; range 21.5–25.0 years).

### Assessment of health-related quality of life

HRQoL was measured using the instrument EQ-5D-3 L and three general questions. The questionnaire in the 16-year follow-up was used to assess HRQoL before the transition, and that from the 24-year follow-up was used to assess HRQoL after the transition. Group-wise comparisons were made.

The instrument EQ-5D-3 L aims to capture physical, mental and social functioning [20, 21]. The EQ-5D-3 L consists of the EQ-5D descriptive system and the EQ visual analogue scale (EQ VAS), and measures the respondent’s health status at the time of completion [20, 21]. The EQ-5D-3 L descriptive system comprises five health attributes (mobility, self-care, usual activities, pain/discomfort and anxiety/depression), and each of the attributes can take on three levels of severity: ‘no problems’, ‘some problems’ and ‘extreme problems’ [20, 22]. EQ VAS consists of a standardised 20 cm long scale with endpoints marked 0 for ‘worst imaginable health score’ and 100 for ‘best imaginable health score’ [22].

Three simple general questions yielded an overall assessment of health, both mental and physical [23, 24]. The questions, response options (dichotomous variables were constructed) and reference groups were (1) ‘How are you feeling?’, Not good/fairly good/good vs. Excellent/very good (used as reference). (2) ‘How healthy do you consider yourself to be?’, Not very healthy/fairly healthy vs. Completely healthy (used as reference). (3) ‘How happy are you with your life right now?’, I am not happy at all/I am not very happy/I am fairly happy vs. I am very happy (used as reference).

### Assessment of explanatory variables

#### Asthma, asthma control and asthma phenotypes

Based on questionnaire data from the 1-, 2-, 4-, 8-, 12-, 16- and 24-year follow-ups, asthma was defined as fulfilling at least two of the following three criteria [25]: Symptoms of wheeze and/or breathing difficulties in the preceding 12 months, ever doctor’s diagnosis of asthma and/or use of asthma medication occasionally or regularly in the preceding 12 months. No asthma at the 24-year follow-up was used as the reference.

The assessment of asthma control was based on questionnaire data from the 16- and 24-year follow-ups, using the modified Global Initiative for Asthma (GINA) definition [26], which included: at least 4 episodes of wheeze, any night-time awakening, activity limitation, and use of a symptom reliever at least 2 times/week in the preceding 12 months. Having at least 3 of 4 symptoms was defined as uncontrolled asthma. Controlled/partly controlled asthma at the 24-year follow-up was used as the reference.

Among those with asthma at the 24-year follow-up, timing of age at onset of disease was categorised into adolescent-onset asthma (fulfilling the criteria for asthma at the 16- or 24-year follow-up, but not at earlier follow-ups), and persistent asthma (fulfilling the criteria for asthma at the 16- or 24-year follow-up and at least one earlier follow-up). Never asthma was (no occasion of asthma at earlier follow-ups) used as the reference.

### Physical activity

Self-reported amount of physical activity in the preceding 12 months was categorised based on the WHO recommendations of physical activity [17]. Adolescents should do ≥ 60 min/day of moderate (e.g., cycling at normal speed or carrying light objects) to vigorous activity (such as lifting heavy weights, aerobics or high-speed cycling), or ≥ 30 min/day of vigorous activity. At the 16-year follow-up, the mean of summer and winter activity was calculated. Adults from 18 years should do ≥ 150 min/week of moderate to vigorous activity or ≥ 75 min/week of vigorous activity. Asthma and fulfilling the physical activity recommendations at the 24-year follow-up was used as the reference.

### Statistical analysis

All analyses were performed cross-sectionally as we compared participants with asthma at the 16- compared to the 24-year follow-up. Descriptive data are presented as numbers (n) and percentages (%) for categorical variables (background characteristics, asthma, asthma control, and physical activity, timing of asthma onset, and the EQ-5D descriptive system), and as medians, interquartile ranges (IQRs) and 95% confidence intervals (CIs) for continuous variables (EQ VAS).

To analyse differences in EQ-5D descriptive system values depending on asthma occurrence, chi-squared and Fisher’s exact tests were used. *p* values of < 0.05 were considered statistically significant.

Quantile regression analyses were performed to investigate associations between the explanatory variables (asthma, asthma control and physical activity) and EQ VAS. Quantile regression analyses were also used for the difference between the 16- and the 24-year follow-ups regarding EQ VAS.

To investigate the associations between the explanatory variables (asthma, asthma control and physical activity) and general health using the three questions ‘How are you feeling?’ (Not good/fairly good/good vs. Excellent/very good), ‘How healthy do you consider yourself to be?’ (Not very healthy/fairly healthy vs. Completely healthy), and ‘How happy are you with your life right now?’ (I am not happy at all/I am not very happy/I am fairly happy vs. I am very happy) at the 24-year follow-up, logistic regression analyses were used.

In the present study, all regression models were adjusted for sex, family history of allergic disease and socio-economic status. Confounders were selected a priori from the previous literature [2]. All analyses were performed with the STATA statistical software (release 14.0; College Station, TX, USA).

## Results

The study population (n = 2,268) had higher proportions of females (55.8% vs. 49.5%) and parents with university education (56.8% vs. 52.9%) than the original cohort (N = 4,089) (Table 1).

**Table 1:** Distribution of selected background characteristics in the BAMSE cohort (N = 4089) and the study population (n = 2268)

Background characteristics	BAMSE cohort N = 4089		Study population n = 2268		
	n	%	n	%	(95% CI)
*Sex*					
Female	2,024	49.5	1,265	55.8	(53.7–57.8)
*Mother’s age at birth*					
< 25 years	319	7.8	156	6.9	(5.9–8.0)
*Parent born outside Sweden*					
Yes	718	21.1	426	20.9	(19.2–22.7)
*Family history of allergic disease*^a^					
Yes	1,200	29.7	701	31.2	(29.3–33.1)
*Parental education*^b^					
University	2,161	52.9	1,289	56.8	(54.8–58.9)
*Parental smoking*^c^					
Yes	855	21.0	458	20.3	(18.7–22.0)

^a^Mother and/or father with doctor’s diagnosis of asthma and asthma medication and/or doctor’s diagnosis of hay fever in combination with reported allergy to furred pets and/or pollen at the time of baseline questionnaire

^b^At least university or college degree/education

^c^Any of the parents smoked at least one cigarette per day at the time of the baseline questionnaire

### Prevalence of asthma, asthma control, physical activity and phenotypes in young adulthood

The prevalence of asthma at the 24-year follow-up was 14.7%. In this group, 83.6% had controlled/partly controlled asthma, 16.4% had uncontrolled asthma, 88.4% fulfilled the WHO recommendations of physical activity and 11.6% did not. Moreover, 62.2% were females and 37.8% males. Further, in the study population 7.7% had adolescent-onset asthma (fulfilling the criteria for asthma at the 16- or 24-year follow-up, but not at earlier follow-ups) and 19.4% had persistent asthma (fulfilling the criteria for asthma at the 16- or 24-year follow-up and at least one earlier follow-up).

### EQ VAS in young adulthood and adolescence

The median value of EQ VAS adjusted for sex, family history of allergic disease and socio-economic status at the 24-year follow-up was 80 in both those with and those without asthma (Table 2). Young adults with uncontrolled asthma had a significantly lower adjusted median EQ VAS score than young adults with controlled/partly controlled asthma (75 vs. 80, *p* = 0.03). Participants with asthma who did not fulfil the WHO recommendations of physical activity had a lower adjusted median EQ VAS than those who did (70 vs. 80, *p* < 0.01). Age of onset of asthma did not influence EQ VAS. When stratified by sex, both females and males with asthma had an adjusted median EQ VAS of 80.

**Table 2:** Distribution of EuroQol visual analogue scale scores at the 24-year follow-up in relation to asthma, asthma control, physical activity and age at onset of asthma (n_tot_ = 2268)

	EuroQol visual analogue scale scores at 24-year follow-up					
	n^g^	Median_crude_ (IQR)	*p* value^e^	n^g^	Median_adj_ (IQR)	*p* value_adj_^f^
*Asthma at 24-year follow-up *						
No	1932	80 (70–90)	Ref.	1876	80 (70–90)	Ref.
Yes^a^	331	80 (70–90)	1.00	323	80 (70–90)	1.00
*With asthma*^a^						
Controlled/partly controlled	274	80 (70–90)	Ref.	268	80 (70–87)	Ref.
Uncontrolled^b^	54	80 (70–90)	1.00	52	75 (70–89)	0.03
Fulfilled physical activity recommendations^c^	250	80 (75–90)	Ref.	246	80 (71–87)	Ref.
Did not fulfil physical activity recommendations	33	70 (65–85)	< 0.01	32	70 (62–87)	< 0.01
*Age at onset of asthma*^d^						
Never asthma	1040	80 (75–90)	Ref.	1023	80 (73–90)	Ref.
Adolescent-onset	111	80 (70–90)	1.00	109	80 (70–90)	1.00
Persistent	275	80 (70–90)	1.00	269	80 (70–90)	1.00

^a^Asthma was defined as fulfilling at least 2 of 3 criteria: symptoms of wheeze and/or breathing difficulties in the preceding 12 months, ever doctor’s diagnosis of asthma and/or use of asthma medication occasionally or regularly in the preceding 12 months

^b^Uncontrolled asthma defined as at least 3 of 4 symptoms: at least 4 episodes of wheeze, any night-time awakening, activity limitation and use of a symptom reliever at least 2 times/week in the preceding 12 months

^c^Self-reported amount of physical activity in the preceding 12 months. Categorised as fulfilling the WHO recommendations where adults from 18 years should do ≥ 150 min/week of moderate to vigorous activity, or ≥ 75 min/week of vigorous activity

^d^Never asthma: no occasion of asthma at earlier follow-ups. Adolescent-onset asthma: asthma at the 16- or 24-year follow-up, but not at earlier follow-ups. Persistent asthma: asthma at the 16- or 24-year follow-up and at least one earlier follow-up (1-, 2-, 4-, 8- or 12-year follow-ups)

^e^*p* values indicate differences between crude median EuroQol visual analogue scale scores and asthma at the 24-year follow-up, obtained using quantile regression

^f^*p* values indicate differences between adjusted median EuroQol visual analogue scale scores and asthma at the 24-year follow-up, obtained using quantile regression. Adjusted for sex, family history of allergic disease and socio-economic status

^g^Missing values due to missing data of EuroQol visual analogue scale scores at 24-year follow-up or one or more of the confounding variables: sex, family history of allergic disease and socio-economic status

**Fig. 1:**
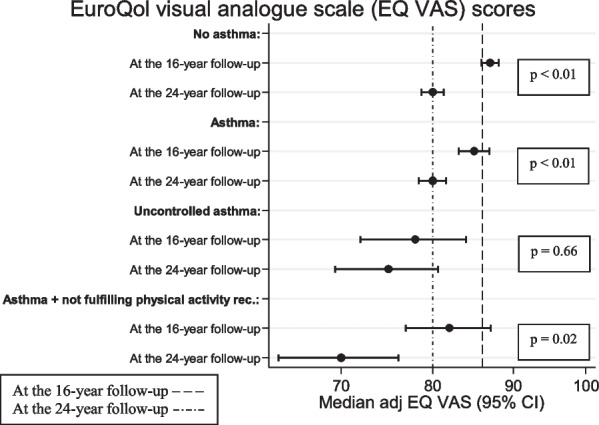
Distribution of EuroQol visual analogue scale scores at the 16- and 24-year follow-ups in relation to asthma, uncontrolled asthma, and asthma and not fulfilling physical activity recommendations (n_tot_ = 2268) ^a^Asthma was defined as fulfilling at least 2 of 3 criteria: symptoms of wheeze and/or breathing difficulties in the preceding 12 months, ever doctor’s diagnosis of asthma and/or use of asthma medication occasionally or regularly in the preceding 12 months ^b^Uncontrolled asthma defined as at least 3 of 4 symptoms: at least 4 episodes of wheeze, any night-time awakening, activity limitation and use of a symptom reliever at least 2 times/week in the preceding 12 months ^c^Self-reported amount of physical activity in the preceding 12 months. Categorised as fulfilling the WHO recommendations, where adolescents should do ≥ 60 min/day of moderate activity to vigorous activity or ≥ 30 min/day of vigorous activity. Adults from 18 years should do ≥ 150 min/week of moderate to vigorous activity or ≥ 75 min/week of vigorous activity ^d^*p* values indicated differences between the 16- and 24-year follow-ups, respectively, and asthma, asthma control and asthma and physical activity regarding median EuroQol visual analogue scale scores obtained using quantile regression. Adjusted for sex, family history of allergic disease and socio-economic status

Compared with at the 16-year follow-up, the adjusted median values of EQ VAS were lower at the 24-year follow-up among participants both with asthma (85 vs. 80, *p* < 0.01) and without asthma (87 vs. 80, *p* < 0.01) (Fig. 1). This was also seen among participants with asthma who were physically inactive (82 vs. 70, *p* = 0.02). For participants with uncontrolled asthma, the adjusted median value of EQ VAS was lower in young adulthood than in adolescence, though the difference was non-significant (78 vs. 75, *p* = 0.66).

### EQ-5D descriptive system in young adulthood and adolescence

In the EQ-5D descriptive system, for the health attribute ‘pain or discomfort’, young adults with asthma reported more problems than peers without asthma (p < 0.01) (Table 3). At the 24-year follow-up, more participants with asthma reported ‘some problems’ or ‘extreme problems’ in the health attributes ‘usual activities’, ‘pain or discomfort’ and ‘anxiety or depression’ than at the 16-year follow-up. Similar increased proportions at the 24-year follow-up were also seen for participants without asthma, for the aforementioned health attributes and also for ‘mobility’ (data not shown).

**Table 3:** Distribution of EQ-5D descriptive system values at the 16- and 24-year follow-ups, respectively, in relation to asthma (n_tot_ = 2,268)

EQ-5D descriptive system values	No asthma at 24-year follow-up n = 1932	Asthma at 24-year follow-up^a^ n = 333	No asthma vs. asthma at 24-year follow-up	Asthma at 16-year follow-up^a^ n = 268	Asthma at 24- versus 16-year follow-up
Health attributes	n (%)	n (%)	*p* value^b^	n (%)	*p* value^c^
*Mobility*					
No problems	1888 (98.0)	325 (97.6)		258 (98.1)	
Some problems	38 (2.0)	8 (2.4)		5 (1.9)	
Extreme problems	1 (0.1)	0 (0)	0.60	0 (0)	0.78
*Self-care*					
No problems	1916 (99.4)	330 (99.4)		259 (98.5)	
Some problems	9 (0.5)	1 (0.3)		1 (0.4)	
Extreme problems	2 (0.1)	1 (0.3)	0.49	3 (1.1)	0.46
*Usual activities*					
No problems	1795 (93.2)	308 (92.5)		256 (97.3)	
Some problems	124 (6.4)	23 (6.9)		6 (2.3)	
Extreme problems	8 (0.4)	2 (0.6)	0.74	1 (0.4)	0.02
*Pain or discomfort*					
No problems	1427 (74.1)	208 (62.5)		192 (73.3)	
Some problems	490 (25.4)	122 (36.6)		70 (26.7)	
Extreme problems	9 (0.5)	3 (0.9)	< 0.01	0 (0.0)	0.01
*Anxiety or depression*					
No problems	1189 (61.7)	199 (59.8)		187 (71.4)	
Some problems	697 (36.2)	120 (36.0)		74 (28.4)	
Extreme problems	41 (2.1)	14 (4.2)	0.09	1 (0.4)	< 0.01

^a^Asthma was defined as fulfilling at least 2 of 3 criteria: symptoms of wheeze and/or breathing difficulties in the preceding 12 months, ever doctor’s diagnosis of asthma and/or use of asthma medication occasionally or regularly in the preceding 12 months

^b^*p* values obtained using Fisher’s exact test to analyse differences between the distribution of EQ-5D descriptive system values for no asthma vs. asthma at the 24-year follow-up

^c^*p* values obtained using Fisher’s exact test to analyse differences between the distribution of EQ-5D descriptive system values for asthma at the 24-year follow-up vs. the 16-year follow-up

When stratified by sex, more females both with and without asthma reported ‘some problems’ or ‘extreme problems’ in the health attribute ‘anxiety or depression’ compared with males (Table 4).

**Table 4:** Distribution of EQ-5D descriptive system values at the 24-year follow-up in relation to asthma and sex (n_tot_ = 2268)

EQ-5D descriptive system values	At 24-year follow-up					
	Females with no asthma ^a^ n = 1057	Males with no asthma ^a^ n = 875	Females vs. males with no asthma	Females with asthma ^a^ n = 207	Males with asthma ^a^ n = 126	Females versus males with asthma
Health attributes	n (%)	n (%)	*p* value^b^	n (%)	n (%)	*p* value^c^
*Mobility*						
No problems	1031 (97.8)	857 (98.2)		201 (97.1)	124 (98.4)	
Some problems	23 (2.2)	15 (1.7)		6 (2.9)	2 (1.6)	
Extreme problems	0 (0.0)	1 (0.1)	0.41	0 (0.0)	0 (0.0)	0.72
*Self-care*						
No problems	1049 (99.5)	867 (99.5)		206 (99.5)	124 (99.2)	
Some problems	4 (0.4)	4 (0.4)		0 (0.0)	1 (0.8)	
Extreme problems	1 (0.1)	1 (0.1)	0.87	1 (0.5)	0 (0.0)	0.61
*Usual activities*						
No problems	984 (93.4)	811 (92.9)		192 (92.8)	116 (92.1)	
Some problems	64 (6.1)	60 (6.9)		14 (6.8)	9 (7.1)	
Extreme problems	6 (0.6)	2 (0.2)	0.42	1 (0.5)	1 (0.8)	1.00
*Pain or discomfort*						
No problems	773 (73.3)	654 (75.0)		121 (58.5)	87 (69.1)	
Some problems	274 (26.0)	216 (24.8)		83 (40.1)	39 (31.0)	
Extreme problems	7 (0.7)	2 (0.2)	0.31	3 (1.5)	0 (0.0)	0.09
*Anxiety or depression*						
No problems	617 (58.5)	572 (65.5)		110 (53.1)	89 (70.6)	
Some problems	409 (38.8)	288 (33.0)		87 (42.0)	33 (26.2)	
Extreme problems	28 (2.7)	13 (1.5)	< 0.01	10 (4.8)	4 (3.2)	0.01

^a^Asthma was defined as fulfilling at least 2 of 3 criteria: symptoms of wheeze and/or breathing difficulties in the preceding 12 months, ever doctor’s diagnosis of asthma and/or use of asthma medication occasionally or regularly in the preceding 12 months

^b^*p* values obtained using chi-squared and Fisher’s exact tests to analyse differences between the distribution of EQ-5D descriptive system values between females and males with no asthma at the 24-year follow-up

^c^*p* values obtained using Fisher’s exact test to analyse differences between the distribution of EQ-5D descriptive system values between females and males with asthma at the 24-year follow-up

### **General health in young adulthood**

When evaluating the question ‘How healthy do you consider yourself to be?’, young adults with asthma had increased odds of feeling not very healthy/fairly healthy compared with young adults without asthma (Table 5). Increased odds of feeling not very healthy/fairly healthy were also seen among young adults with uncontrolled asthma compared with young adults with controlled/partly controlled asthma, as well as among the participants with asthma who did not fulfil the WHO recommendations of physical activity compared with peers who did. Moreover, participants with either adolescent-onset or persistent asthma had increased odds of feeling not very healthy/fairly healthy compared with the never asthma group (Table 5). For the same question, ‘How healthy do you consider yourself to be?’ when.

**Table 5:** General health at the 24-year follow-up in relation to asthma, asthma control, physical activity and age at onset of asthma (n_tot_ = 2,268)

Questions asked	n	General health at 24-year follow-up		
		How are you feeling?	How healthy do you consider yourself to be?	How happy are you with your life right now?
Response options		Not good/fairly good/good versus Excellent/very good	Not very healthy/fairly healthy versus Completely healthy	I am not happy at all/I am not very happy/I am fairly happy versus I am very happy
		OR_adj_^e^ (95% CI)	OR_adj_^e^ (95% CI)	OR_adj_^e^ (95% CI)
*Asthma at the 24-year follow-up*				
No	1876	Ref.	Ref.	Ref.
Yes^a^	323	1.2 (1.0–1.6)	2.1 (1.7–2.7)	1.1 (0.9–1.4)
Controlled/partly controlled	268	Ref.	Ref.	Ref.
Uncontrolled^b^	52	1.7 (0.9–3.1)	3.1 (1.5–6.1)	1.3 (0.7–2.5)
Fulfilled physical activity recommendations^c^	246	Ref.	Ref.	Ref.
Did not fulfil recommended physical activity recommendations	32	2.0 (0.9–4.3)	2.6 (1.1–6.0)	2.8 (1.1–7.1)
*Age at onset of asthma*^d^				
Never asthma	1023	Ref.	Ref.	Ref.
Adolescent-onset	109	1.7 (1.2–2.6)	1.7 (1.1–2.5)	1.2 (0.8–1.8)
Persistent	269	1.3 (1.0–1.7)	1.9 (1.5–2.6)	1.1 (0.8–1.4)

^a^Asthma was defined as fulfilling at least 2 of 3 criteria: symptoms of wheeze and/or breathing difficulties in the preceding 12 months, ever doctor’s diagnosis of asthma and/or use of asthma medication occasionally or regularly in the preceding 12 months

^b^Uncontrolled asthma defined as at least 3 of 4 symptoms: at least 4 episodes of wheeze, any night-time awakening, activity limitation and use of a symptom reliever at least 2 times/week in the preceding 12 months

^c^Self-reported amount of physical activity in the preceding 12 months. Categorised as fulfilling the WHO recommendations where adults from 18 years should do ≥ 150 min/week of moderate to vigorous activity or ≥ 75 min/week of vigorous activity

^d^Never asthma: no occasion of asthma at earlier follow-ups. Adolescent-onset asthma: asthma at the 16- or 24-year follow-up, but not at earlier follow-ups. Persistent asthma: asthma at the 16- or 24-year follow-up and at least one earlier follow-up (1-, 2-, 4-, 8- or 12-year follow-ups)

^e^Results obtained with logistic regression. Adjusted for sex, family history of allergic disease, and socio-economic status

stratifying by sex, females with asthma had increased odds of feeling not very healthy/fairly healthy compared with males with asthma (OR_adj_ 2.0, 95% CI 1.2–3.1).

Evaluating the question ‘How are you feeling?’ revealed that the group with adolescent-onset asthma had increased odds of feeling not good/fairly good/good (OR_adj_ 1.7, 95% CI 1.2–2.6) compared with the never asthma group (Table 5). For the question ‘How happy are you with your life right now?’, young adults with asthma who did not fulfil the WHO recommendations of physical activity had increased odds of responding ‘I am not happy at all/I am not very happy/I am fairly happy’ compared with peers who did fulfil the recommendations.

## Discussion

We investigated HRQoL among young people with asthma during the transition from adolescence to young adulthood, based on a large sample, with data of asthma symptoms up to approximately 24 years of age in the population-based BAMSE birth cohort. Our results revealed that HRQoL was lower in young adulthood, after the transition, than in adolescence. Furthermore, young adults with asthma having uncontrolled disease or who were physically inactive appeared to be particularly vulnerable.

The general decrease in HRQoL with age in the present study may be a part of emerging adulthood, which is characterised by changing life circumstances. For instance, as adolescents move through the educational system, they are subjected to greater academic demands and expectations [27]. School satisfaction has been considered a fundamental domain for the understanding of students’ quality of life. An international comparative survey showed that perceived school pressure tends to increase in the transition through adolescence, and to differ with gender, with older girls reporting the highest levels of school pressure [28]. Further, another international survey examined trends in school pressure and school satisfaction by gender among 15-year-old students between 2002 and 2018 [27]. They found that school satisfaction tended to increase over the period among boys, whereas school pressure increased among girls. These sex differences were supported by our results.

Our results are in accordance with recent results from the PIAMA birth cohort, where decreased mental well-being and general health were seen from age 17 to 20 years among participants both with and without asthma [29]. In the present study, HRQoL decreased among young adults with asthma, and more problems were reported in three out of five health attributes in young adulthood compared with in adolescence. A reason for this could be that the young adults are supposed to self-manage their asthma [30]. This can be challenging; we saw in our previous study that young adults with asthma felt left out of the system during the transition from paediatric to adult healthcare and did not know where to turn in adult healthcare [31]. Providently, the European Academy of Allergy and Clinical Immunology recently developed clinical practice guidelines to provide evidence-based recommendations for healthcare professionals to support the transitional care of adolescents and young adults with asthma [6]. The recommendations include identifying and managing issues impacting HRQoL. In contrast to our study, one of the few published studies, designed to evaluate HRQoL of adolescents with asthma when they were transferred from paediatric to adult healthcare, found that HRQoL among adolescents improved between the ages of 16 and 21 years [32]. This study was conducted at a children’s hospital, and the authors reasoned that one possible explanation for the improvement could be improved asthma control during this period [33].

In the present study, uncontrolled asthma had an impact on HRQoL among young adults. This confirms results in prior literature and implies that patients with uncontrolled asthma should be observed more closely [26, 34]. Moreover, a recent cross-sectional study found that medication adherence also correlates with better HRQoL in adolescence [35]. Recent results from our BAMSE cohort show that controller medication adherence (i.e., refilling a prescription within 18 months) tends to be low in young adults with asthma (60%) [18]. A recent meta-analysis indicates that non-adherence to inhaled corticosteroids is a significant problem during emerging adulthood, a potentially challenging transition [36]. Therefore, a significant proportion of patients is not benefiting from effective asthma treatment in early adulthood, leading to a high prevalence of uncontrolled asthma. This highlights the need to address non-adherence in this population. Further potential factors related to HRQoL is body mass index (BMI), as it is known to be associated with asthma, reflecting lifestyle differences [37]. However, in our study, the young adults with asthma who did not fulfil the WHO recommendations of physical activity per week had increased odds of considering themselves not very healthy or fairly healthy and had a lower HRQoL compared with their peers who did fulfil the recommendations. A recent systematic review evaluating the effects of physical activity on asthma outcomes showed that most studies suggest that physical activity improves HRQoL, as well as asthma control, lung function parameters and inflammatory markers among adults with an asthma diagnosis [38]. However, exercise-induced bronchoconstriction (EIB) is common in patients with asthma and can be one factor negatively affect motivation and participation in physical activity [26, 39]. Given the negative impact of EIB, early detection of EIB is an important consideration for healthcare providers, resulting in increased physical activity levels throughout life, improved cardiovascular conditioning, reduced rates of obesity and better HRQoL [40]. However, since information on physical activity and asthma were collected at the same time, no conclusions on the temporal relationships between these variables could be drawn from our data. These results may though be relevant in clinical practice as support for the benefits of non-pharmacological interventions [41]. Another comorbid condition is gastroesophageal reflux disease (GERD), where a recent systematic review found that GERD and asthma exacerbation are weakly associated [42]. However, it would be interesting to investigate potential association between HRQoL among participants with asthma and GERD, as both asthma and GERD have a high prevalence in westernized countries.

In the present study, both young adults with adolescent-onset and those with persistent asthma had increased odds of not considering themselves healthy compared with participants without asthma. These results are supported by a recent systematic literature review which aimed to understand the challenges faced by adolescents and young adults with asthma and allergic conditions. The review found that onset of disease in adolescence was linked to impairment of HRQoL [1].

### Strengths and limitations

One important strength of the present study was the prospective data collection from birth up to young adulthood, which enabled us to assess age at onset of asthma. Other strengths included the population-based design and the large and well-characterised study sample of the BAMSE cohort. Further, the EQ-5D is the most popular generic instrument for measuring HRQoL in patients with asthma [43]. A recent systemic review assessed the evidence on the validity and responsiveness of five commonly used preference-based instruments, including EQ-5D [44]. Based on sixteen reviews, covering more than 180 studies, results were heavily skewed towards EQ-5D, with significantly fewer studies investigating other instruments. There was evidence that EQ-5D was generally valid and responsive. Still, a variety of HRQoL instruments was used in relation to asthma, e.g., the Short Form 36 Health, the DISABKIDS asthma module questionnaire, the SF-6D and the KIDSCREEN-10 [12, 13, 45, 46]. Since asthma is an episodic disease, the ability of the generic EQ-5D instrument to reflect the full impact ‘today’ could be discussed, e.g., whether the measure can capture the impact of exacerbations between episodes; it may miss clinically important changes in asthma control [47]. Although greater sensitivity may be demonstrated with a disease-specific instrument, those may lack the ability to compare utility values across diseases and may also miss side effects and comorbidities [9].

The study population consisted of participants who responded to the questionnaire and participated in the 24-year clinical examination, and the majority had data of EQ VAS at both the 16- and the 24-year follow-ups. Thus, the decrease in EQ VAS is not likely to be explained by loss to follow-up.

Further, asthma control was based on information for the 12 months preceding each questionnaire, but the GINA guidelines base asthma control on information from the last 4 weeks [26]. This could lead to an overestimation in our data of the proportion of adolescents and young adults with uncontrolled asthma.

## Conclusion

HRQoL is lower in young adulthood than in adolescence. Young adults with asthma having uncontrolled disease or who are physically inactive appear to be particularly vulnerable. Therefore, healthcare providers should give support for better asthma control and address physical activity in asthma management during the transition process.

## Data Availability

The datasets generated and/or analysed during the current study are not publicly available due to the dataset containing sensitive personal data but are available from the corresponding author on reasonable request and with permission of Karolinska Institutet.

## Abbreviations

95% CI: 95% Confidence interval
BAMSE: Barn/Child, Allergy, Milieu, Stockholm, Epidemiology
BMI: Body mass index
EIB: Exercise-induced bronchoconstriction
EQ VAS: EuroQol visual analogue scale
GERD: Gastroesophageal reflux disease
GINA: Global Initiative for Asthma
HRQoL: Health-related quality of life
IQR: Interquartile range
OR_adj_: Adjusted odds ratio
WHO: World Health Organization
